# Effect of Nonpharmaceutical Interventions on Transmission of Severe Acute Respiratory Syndrome Coronavirus 2, South Korea, 2020

**DOI:** 10.3201/eid2610.201886

**Published:** 2020-10

**Authors:** Sukhyun Ryu, Seikh Taslim Ali, Cheolsun Jang, Baekjin Kim, Benjamin J. Cowling

**Affiliations:** Konyang University College of Medicine, Daejeon, South Korea (S. Ryu, C. Jang, B. Kim);; The University of Hong Kong, Hong Kong, China (S.T. Ali, B.J. Cowling)

**Keywords:** 2019 novel coronavirus disease, coronavirus disease, COVID-19, severe acute respiratory syndrome coronavirus 2, SARS-CoV-2, viruses, respiratory infections, zoonoses, transmissibility, South Korea, public health measures

## Abstract

We analyzed transmission of coronavirus disease outside of the Daegu-Gyeongsangbuk provincial region in South Korea. We estimated that nonpharmaceutical measures reduced transmissibility by a maximum of 34% without resorting to a strict lockdown strategy. To optimize epidemic control, continuous efforts to monitor the transmissibility are needed.

Infection with severe acute respiratory syndrome coronavirus 2 (SARS-CoV-2) was identified in South Korea on January 20, 2020 (*1*). By April 21, 2020, a total of 10,683 cases of coronavirus disease (COVID-19) in South Korea had been confirmed and 237 persons had died. (*2*) (Figure 1, panel A). A large number of COVID-19 cases and deaths resulted from superspreading events in the Daegu-Gyeongsangbuk provincial region of South Korea (Figure 1, panel B). On February 23, 2020, during the early phase of the outbreak as the number of COVID-19 cases increased, public health authorities in South Korea raised the infectious disease alert to its highest level (*3*). Subsequently, enhanced screening and testing in the community (operation of drive-through screening centers and designation of private hospitals where COVID-19 screening testing was available) were implemented (*4*,*5*).

**Figure 1:**
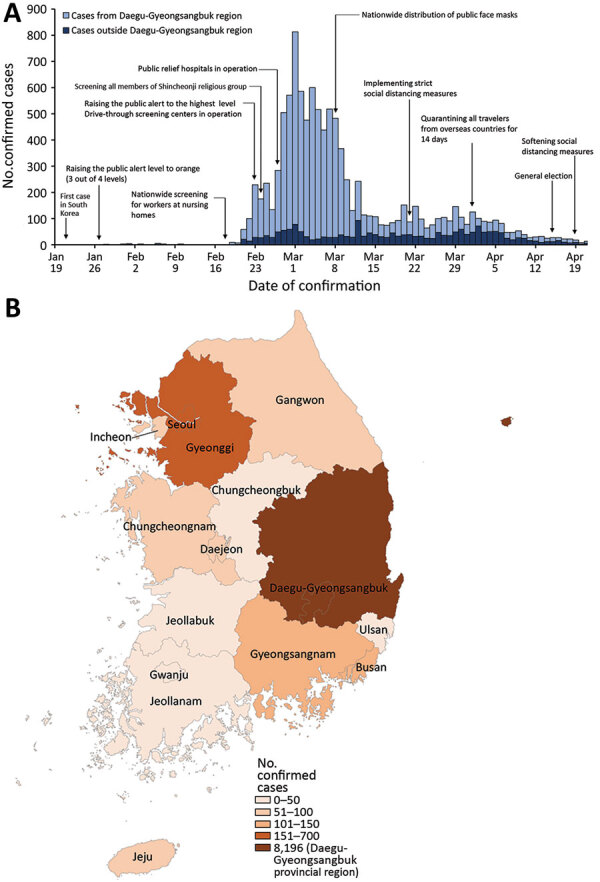
Timeline (A) and geographic distribution (B) of laboratory-confirmed cases of coronavirus disease in South Korea as of April 21, 2020. *Daegu-Gyeongsanbuk provincial region.

On April 19, 2020, public health authorities in South Korea started to relax social distancing measures, which had been implemented on March 21, 2020; as of April 21, 2020, the COVID-19 epidemic in South Korea had been contained. Recent studies have examined how public health interventions can contain COVID-19 outbreaks (*6*,*7*). However, in the absence of information on public health measures against transmission of SARS-CoV-2 in South Korea, we estimated the transmissibility of SARS-CoV-2 and evaluated the effects of the public health measures implemented outside the Daegu-Gyeongsangbuk provincial region in South Korea. 

## The Study

We collected data published by local public health authorities in South Korea, including the city or provincial departments of public health. The data comprised date of exposure; date of illness onset; and the source of infection, including contact history and demographic characteristics (e.g., patient birth year and sex). We extracted these line list data of cases by using an electronic data-extraction form. We divided the study into 2 periods, before and after the declaration of highest public alert: period 1 (January 20–February 23, 2020) and period 2 (February 24–April 21, 2020). We restricted our analysis to all other regions in South Korea that excluded Daegu-Gyeongsangbuk provincial region, where there were superspreading events and the data have not been made publicly available (*8*). Over the entire 3-month study period (January 20–April 21, 2020), data were collected for 2,023 cases, which accounted for 98% of the 2,066 reported cases from the South Korea Ministry of Health and Welfare.

The median case-patient age was 42 (range 1–102) years, and 820 (41%) case-patients were male (Table). We analyzed the statistical differences in patient age and sex between periods 1 and 2 by using the χ^2^ test but did not identify any significant differences. The proportion of cases imported from Daegu-Gyeongsangbuk provincial regions was 31% in period 1 and decreased to 5% in period 2. However, during the same periods, the proportion of cases imported from abroad and cases occurring in large clusters increased from 8% to 30%.

**Table:** Demographic characteristics of 2,023 persons with confirmed cases of coronavirus disease, from publicly available data, South Korea, outside of Daegu-Gyeongsangbuk provincial region on April 21, 2020*

Characteristic	**All, no. (%)**	**Period 1, no. (%)†**	**Period 2, no. (%)‡**
Age group, y			
0–19	123 (6)	11 (5)	112 (6)
20–39	715 (35)	104 (50)	611 (34)
40–59	619 (31)	50 (24)	569 (31)
60–79	295 (15)	37 (18)	258 (14)
>80y	50 (3)	6 (3)	44 (2)
Unknown	221 (11)	0	221(12)
Sex			
M	820 (41)	107 (56)	713 (39)
F	953 (47)	100 (43)	853 (47)
Unknown	250 (12)	1 (1)	249 (14)
Type of transmission§			
Local	892 (44)	116 (55)	776 (43)
Imported from Daegu-Gyeongsangbuk	155 (8)	65 (31)	90 (5)
Imported from abroad	552 (27)	16 (8)	536 (30)
Cases occurring in large clusters	424 (21)	11 (5)	413 (23)

*Assignment to period was based on date of symptom onset. If cases were asymptomatic or date of symptom onset date was not reported, we used the date of case confirmation.  †Jan 20–Feb 23, 2020; n = 208. ‡Feb 24–Apr 21, 2020; n = 1,815. §Source of infection is provided for all cases; if not identified, we considered the case to have occurred by local transmission.

We analyzed the time interval between illness onset and laboratory confirmation for 818 symptomatic case-patients. We estimated the mean time interval from symptom onset to confirmation of COVID-19 during periods 1 and 2 by fitting 3 parametric distributions (Weibull, gamma, and log-normal) and based our selection of best fit on the Akaike information criterion (*9*). We found the log-normal distribution to be the best fit for this time interval, with a mean of 4.6 (95% CI 0.0–12.4) for period 1 and a substantial reduction to 3.4 (0.0–9.0) for period 2. 

To estimate the incubation period, we analyzed data from 181 case-patients for whom precise contact history with other confirmed case-patients was known. The incubation period was estimated by fitting 3 parametric distributions and best fitted by the log-normal distribution; the overall estimated median incubation period was 4.7 (95% CI 0.1–15.6) days (Appendix). We identified 44 clusters of infection and 79 case-patients who had had clear exposure to only 1 index case-patient among these clusters (Appendix). Overall, serial intervals were negative for 8 of the 79 transmission pairs. We estimated the serial interval distribution by fitting a normal distribution to all 79 observations (*10*). We estimated a mean (± SD) serial interval to be 3.9 (± 4.2) days (Appendix).

**Figure 2:**
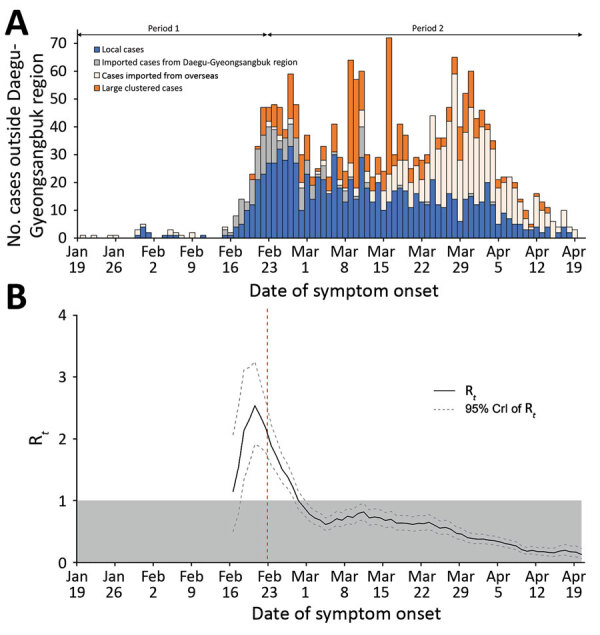
Incidence and estimated daily effective reproductive number (R*_t_* ) of coronavirus disease in regions outside of Daegu-Gyeongsanbuk provincial region, South Korea, as of April 21, 2020. A) The epidemic curve shows the daily number of patients with confirmed cases and symptom onset. For case-patients who did not report any symptoms on the date of case confirmation (n = 1,205 cases; 60% of total), the date of confirmation was plotted instead. B) Daily estimated R*_t_* and 95% CrI of R*_t_*; shading indicates the area below the epidemic threshold of R*_t_* = 1. The vertical dashed line indicates the start of the highest public alert on February 23, 2020. CrI, credible interval.

In mid-February 2020, the number of cases rapidly increased; the largest proportion of cases was among persons who had been infected in Daegu-Gyeongsangbuk provincial region and traveled to other regions of South Korea (Figure 2, panel A). To investigate the effectiveness of nonpharmaceutical interventions implemented in South Korea (Appendix), we estimated the instantaneous effective reproduction number (R*_t_*), a real-time measure of transmission intensity, from daily onset of cases and our estimated serial interval distribution by using the EpiEstim package in R (*11*,*12*). R*_t_* is defined as the mean number of secondary infections per primary case with illness onset at time *t*; R*_t_*<1 indicates that the epidemic is under control.

We present the daily estimates of R*_t_* from February 16, 2020, because the stable estimate of R*_t_* was not available due to the low number of confirmed cases (Figure 2, panel B). At the end of period 1, on February 21, mean R*_t_* peaked at 2.85 (95% credible interval [CrI] 2.02–3.87) and then started to decline faster to <1 by February 29. R*_t_* further declined and remained at <1 during the rest of period 2, indicating the potential effect of nonpharmaceutical interventions implemented over time (Figure 2, panel B). Specifically, mean R*_t_* was 2.23 (CrI 2.05–2.40) before the 1-week period when the declared public alert was at the highest level and reduced to 1.48 (CrI 1.36–1.60) in the following 1-week period, corresponding to a 33.6% (95% CI 23.46%–43.44%) reduction in transmissibility. Similarly, along with the high public alert, the implementation of strict social distancing measures on March 12, 2020, was associated with an R*_t_* reduction of an additional 9.28% (95% CI 6.80%–11.75%).

## Conclusions

Combined nonpharmaceutical interventions, including enhanced screening and quarantining of persons with suspected and confirmed cases and social distancing measures, were implemented over time. Our results suggest that those interventions, without a lockdown, reduced the transmissibility of SARS-CoV-2 in regions outside of the Daegu-Gyeongsangbuk provincial region, in South Korea.

Our study has some limitations. First, in our analysis of the changes of transmissibility of SARS-CoV-2, we did not include the large clustered cases reported as superspreading events because in these large clusters, the reporting date may not be a good proxy for the date of infection and would overestimate R*_t_*(*13*). Second, it is uncertain how many cases were still undetected. This proportion may potentially mislead the actual time trends of number of infections in the population. Third, we based our estimation of time delay on self-reported data, which are not free from reporting (recall) bias. Fourth, government-generated data, including dates of symptom onset, were not available; therefore, we retrieved online case reports, which could have resulted in some inaccuracies in the information used in our analyses. However, the daily numbers of confirmed cases from the collected line list we used was similar to the numbers in the official daily reports (Appendix).

Our findings suggest that the nonpharmaceutical interventions implemented in South Korea during the COVID-19 outbreak effectively reduced virus transmissibility and suppressed local spread. However, the population of South Korea is still susceptible to further outbreaks or epidemic waves. Because social distancing measures will be relaxed while opportunities for importation of infections from abroad continue, ongoing monitoring of the effective reproductive number can provide relevant information to help policymakers control a potential second wave of COVID-19.

Appendix Additional data from study of effect of nonpharmaceutical interventions on transmission of severe acute respiratory syndrome coronavirus 2, South Korea, 2020.
