# Supplementary material for: Effect of Nonpharmaceutical Interventions on Transmission of Severe Acute Respiratory Syndrome Coronavirus 2, South Korea, 2020
Source: Emerg Infect Dis. 2020 Oct;26(10):2406–10. doi: 10.3201/eid2610.201886 (PMC7510738; doi:10.3201/eid2610.201886)
Supplement: Appendix — Additional data from study of effect of nonpharmaceutical interventions on transmission of severe acute respiratory syndrome coronavirus 2, South Korea, 2020. [file 20-1886-Techapp-s1.pdf]

Article DOI: https://doi.org/10.3201/eid2610.201886

Effect of Nonpharmaceutical Interventions on Transmission of Severe Acute Respiratory Syndrome Coronavirus 2, South Korea, 2020

Appendix

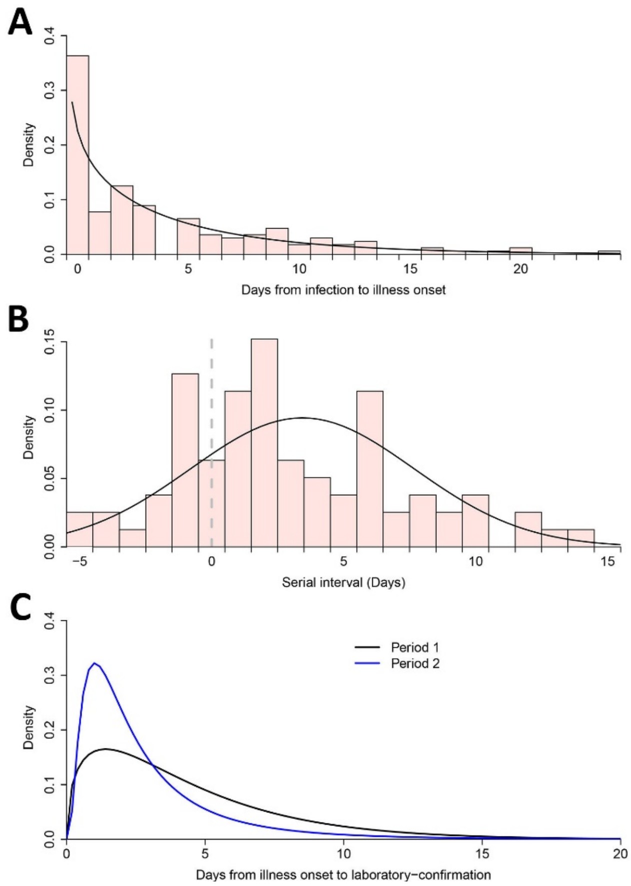

Appendix Figure 1. Estimates of epidemiological distributions, COVID-19, South Korea. A) Distribution of incubation period among 181 laboratory-confirmed cases. The line is best fitted by a gamma distribution.

B) Distribution of serial interval among 79 cases in 44 clusters. The line indicates a fitted normal distribution. C) Distribution of symptom onset to laboratory-confirmation divided by two periods of the epidemic in South Korea. The black line is the estimate during period-1(before February 23, 2020), and the blue line is the estimate during period-2 (on or after February 24, 2020). Both lines indicate a fitted log-normal distribution.

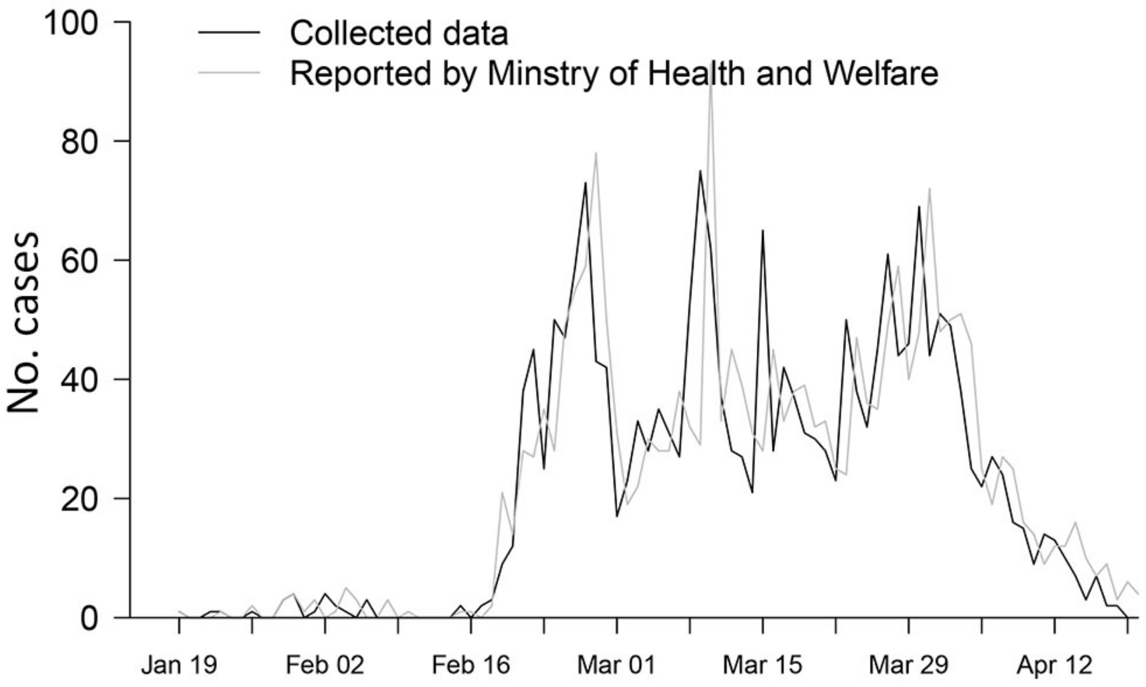

Appendix Figure 2. Daily number of laboratory-confirmed cases from the collected data and reported data by the Korean Ministry of Health and Welfare outside of Daegu-Gyeongsangbuk provincial region in South Korea. The black line indicates the collected data used in this study, and the gray line indicates the daily number of confirmed cases reported from the central government in South Korea.

Appendix Table 1. The dates of symptom onset among 79 cases in 44 clusters of infection. We retrieved the line-list data of the cases by using electronic data-extraction form (MS excel software). The column of this excel spread sheet presents the variables, including the date of exposure, date of illness onset, source of infection, demographic characteristics including birth year and gender of the cases.

Number of cluster	Index case	Symptom onset	Sex	Age	Source ID from local public health authorities
1	1	February 18, 2020	Female	26	Busan #11
1	0	February 19, 2020	Male	29	Busan #39
1	0	February 23, 2020	Female	32	Busan #56
2	1	February 21, 2020	Female	57	Busan #2
2	0	February 20, 2020	Female	25	Busan #5
2	0	February 21, 2020	Female	82	Busan #6
2	0	February 22, 2020	Female	44	Busan #25
2	0	February 29, 2020	Female	83	Busan #87
3	1	February 21, 2020	Male	19	Busan #15
3	0	February 26, 2020	Male	16	Busan #62
3	0	February 28, 2020	Male	19	Busan #82
4	1	February 21, 2020	Female	25	Busan #59
4	0	February 26, 2020	Male	25	Busan #65
4	0	February 24, 2020	Female	51	Busan #58
5	1	February 17, 2020	Male	25	Busan #13
5	0	February 20, 2020	Female	56	Busan #20
6	1	February 22, 2020	Female	62	Busan #78
6	0	March 1, 2020	Female	36	Busan #81
7	1	February 17, 2020	Female	28	Busan #36
7	0	February 23, 2020	Male	27	Busan #54
7	0	February 26, 2020	Female	18	Busan #70
7	0	March 2, 2020	Female	40	Busan #85
8	1	February 28, 2020	Male	79	Busan #71
8	0	March 9, 2020	Male	50	Busan #92
8	0	March 12, 2020	Male	50	Busan #98
9	1	March 6, 2020	Female	68	Busan #100

Number of cluster	Index case	Symptom onset	Sex	Age	Source ID from local public health authorities
9	0	March 9, 2020	Male	73	Busan #97
10	1	February 19, 2020	Male	25	Busan #57
10	0	February 23, 2020	Female	65	Busan #60
11	1	February 6, 2020	Female	82	Seoul #14
11	0	February 15, 2020	Male	82	Seoul #13
12	1	February 27, 2020	Female	42	Seoul #140
12	0	February 29, 2020	Female	61	Seoul #164
13	1	February 24, 2020	Female	60	Seoul #38
13	0	March 6, 2020	Male	65	Seoul #117
14	1	March 12, 2020	Male	26	Seoul #266
14	0	March 19, 2020	Female	29	Seoul #297
14	0	March 18, 2020	Female	55	Seoul #298
15	1	March 11, 2020	Female	30	Ulsan #29
15	0	March 12, 2020	Male	30	Ulsan #30
16	1	March 16, 2020	Female	30	Ulsan #31
16	0	March 16, 2020	Male	30	Ulsan #36
17	1	March 17, 2020	Female	26	Seoul #304
17	0	March 20, 2020	Male	61	Seoul #320
18	1	March 19, 2020	Female	4	Seoul #311
18	0	March 22, 2020	Female	38	Seoul #314
19	1	February 27, 2020	Female	55	Suwon #13
19	0	February 29, 2020	Male	42	Suwon #16
20	1	March 7, 2020	Male	24	Suwon #17
20	0	March 9, 2020	Male	10	Suwon #18
21	1	March 20, 2020	Male	20s	Suwon #23
21	0	March 22, 2020	Male	50s	Suwon #24
21	0	March 22, 2020	Female	20s	Suwon #26
22	1	March 27, 2020	Female	55	Suwon #44
22	0	March 31, 2020	Male	42	Suwon #45
23	1	March 21, 2020	Female	30	Yongin #41
23	0	March 22, 2020	Male	57	Yongin #43
24	1	March 14, 2020	Male	49	Yongin #29
24	0	March 19, 2020	Female	49	Yongin #27

Number of cluster	Index case	Symptom onset	Sex	Age	Source ID from local public health authorities
24	0	March 27, 2020	Female	77	Yongin #47
25	1	March 20, 2020	Female	68	Yongin #34
25	0	March 21, 2020	Male	44	Yongin #35
25	0	March 26, 2020	Female	44	Yongin #44
26	1	March 25, 2020	Not available	20s	Uijeongbu #18
26	0	March 30, 2020	Not available	20s	Uijeongbu #19
26	0	April 1, 2020	Not available	50s	Uijeongbu #22
27	1	February 20, 2020	Female	40s	Sejong #2
27	0	February 22, 2020	Female	40s	Sejong #6
27	0	February 27, 2020	Female	20s	Sejong #3
27	0	February 22, 2020	Female	50s	Sejong #4
27	0	March 1, 2020	Female	40s	Sejong #5
28	1	February 21, 2020	Female	47	Gyeongnam #5
28	0	February 18, 2020	Male	21	Gyeongnam #7
28	0	February 24, 2020	Male	16	Gyeongnam #29
28	0	February 25, 2020	Male	41	Gyeongnam #42
29	1	April 2, 2020	Female	39	Gyeongnam #109
29	0	April 4, 2020	Male	9	Gyeongnam #110
30	1	February 23, 2020	Female	72	Namyangju #2
30	0	February 23, 2020	Male	77	Namyangju #3
31	1	March 28, 2020	Female	20s	Jeju #9
31	0	April 1, 2020	Male	30s	Jeju #11
32	1	April 9, 2020	Male	60s	Guri #5
32	0	April 6, 2020	Female	61	Pocheon #12
32	0	April 12, 2020	Female	60s	Pocheon #14
33	1	March 23, 2020	Female	46	Pyeongtaek #19
33	0	March 22, 2020	Female	78	Pyeongtaek #26
33	0	March 25, 2020	Male	79	Pyeongtaek #27
33	0	March 23, 2020	Female	34	Pyeongtaek #28
33	0	March 26, 2020	Female	32	Pyeongtaek #29
33	0	April 3, 2020	Male	47	Pyeongtaek #35
33	0	April 6, 2020	Male	54	Pyeongtaek #38
34	1	March 25, 2020	Male	9	Pyeongtaek #36

Number of cluster	Index case	Symptom onset	Sex	Age	Source ID from local public health authorities
34	0	March 19, 2020	Female	49	Pyeongtaek #39
35	1	February 24, 2020	Female	40s	Gwangmyeong #2
35	0	February 28, 2020	Male	49	Gwangmyeong #3
35	0	March 2, 2020	Male	11	Gwangmyeong #4
36	1	April 6, 2020	Not available	60s	Uijeongbu #28
36	0	April 6, 2020	Not available	60s	Uijeongbu #29
36	0	April 9, 2020	Male	60	Gwangju #15
36	0	April 9, 2020	Female	56	Gwangju #16
37	1	February 25, 2020	Female	49	Gunpo #2
37	0	March 3, 2020	Male	78	Ansan #1
37	0	March 3, 2020	Female	73	Ansan #3
38	1	March 3, 2020	Female	48	Gwangmyeong #2
38	0	February 28 2020	Male	49s	Gwangmyeong #3
38	0	March 2, 2020	Male	11	Gwangmyeong #4
39	1	February 18, 2020	Male	36	Chungbuk #2
39	0	February 18, 2020	Female	35	Chungbuk #3
39	0	March 4, 2020	Female	57	Chungbuk #14
40	1	March 8, 2020	Female	42	Chungnam #107
40	0	March 6, 2020	Male	1	Chungnam #108
41	1	March 6, 2020	Male	38	Chungnam #94
41	0	March 6, 2020	Female	32	Chungnam #95
41	0	March 6, 2020	Female	3	Chungnam #96
42	1	April 7, 2020	Female	42	Seongnam #120
42	0	April 10, 2020	Male	1	Seongnam #122
43	1	February 21, 2020	Male	21	Busan #29
43	0	February 24, 2020	Male	19	Busan #42
43	0	February 21, 2020	Female	51	Busan #43
44	1	February 21, 2020	Female	25	Busan #59
44	0	February 24, 2020	Female	51	Busan #58
44	0	February 21, 2020	Female	44	Busan #61
44	0	February 28, 2020	Female	31	Busan #63

Appendix Table 2. Key non-pharmaceutical interventions in South Korea (most of the interventions)

Starting date	Interventions	Source
**Travel-related measures**		
February 3, 2020	Barred of entry of foreign travelers from Hubei province, China	http://overseas.mofa.go.kr/ru-ko/brd/m_7329/view.do?seq=1345588&srchFr=&srchTo=&srchWord=&srchTp=&multi_itm_seq=0&itm_seq_1=0&itm_seq_2=0
February 23, 2020	Recommended travel restriction in Daegu City<br>- Recommended avoiding social gathering and refraining from going out in Daegu city	http://ncov.mohw.go.kr/tcmBoardView.do?contSeq=353064
March 9, 2020	Barred of entry of foreign travelers from Japan	http://overseas.mofa.go.kr/jp-ko/brd/m_1083/view.do?seq=1343492
April 1, 2020	Implemented 14-day mandatory quarantine to all travellers entering Korea from abroad	http://overseas.mofa.go.kr/nl-en/brd/m_6971/view.do?seq=761545
**Case-based measures**		
February 17, 2020	Implemented screening test for COVID-19 for the health care workers at all nursing home	https://www.cdc.go.kr/board/board.es?mid=a30402000000&bid=0030&act=view&list_no=366586&tag=&nPage=1
February 23, 2020	Launched nationwide drive-through screening centers<br>- Operated 58 roadside screening sites to timely identify the infected cases in the community as of May 11, 2020	https://jkms.org/DOIx.php?id=10.3346/jkms.2020.35.e123<br>https://www.mohw.go.kr/react/popup_200128_4.html
February 25, 2020	Initiated screening all Sincheonji religious group members<br>- As the Sincheonji religious group occupied a large portion of COVID-19 cases in Korea, Korean public health authorities initiated the screening program for this group members (ca. 0.2 million)	http://www.korea.kr/news/pressReleaseView.do?newsId=156377318

Starting date	Interventions	Source
February 27, 2020	Designated private hospitals as public relief hospital in nationwide<br>- Operated 339 public relief hospitals where COVID-19 screening test is available as of May 11, 2020	http://ncov.mohw.go.kr/tcmBoardView.do?brdId=&brdGubun=&dataGubun=&ncvContSeq=353184&contSeq=353184&board_id=140&gubun=BDJ<br>https://www.mohw.go.kr/react/popup_200128_4.html
March 9, 2020	Implemented nationwide screening the elderly at nursing home	https://www.gov.kr/portal/ntnadmNews/2120440
**Community measures**		
February 23, 2020	Raised the infectious disease alert to the highest level	https://www.cdc.go.kr/board/board.es?mid=a20501000000&bid=0015&act=view&list_no=366324&tag=&nPage=1
February 23, 2020	Postponed school opening for new semester<br>- School breaks were extended nationwide until the notification by Korean Ministry of Education	https://www.moe.go.kr/boardCnts/view.do?boardID=294&boardSeq=79829&lev=0&searchType=S&statusYN=W&page=1&s=moe&m=020402&opType=N
March 9, 2020	Distributed public face masks<br>- Evenly provided the face masks to the public through the public channels to prevent stockpiling	https://www.mfds.go.kr/brd/m_99/view.do?seq=44020&srchFr=&srchTo=&srchWord=&srchTp=&itm_seq_1=0&itm_seq_2=0&multi_itm_seq=0&company_cd=&company_nm=&page=1
March 22, 2020	Implemented social distancing measures<br>- Recommended canceling any social event, avoiding social gathering and refraining from going out in nationwide	http://ncov.mohw.go.kr/shBoardView.do?brdId=2&brdGubun=27&ncvContSeq=1385#
April 20, 2020	Softened social distancing measures<br>- Relaxed the measures for religious gathering, playing sports, etc.	http://www.mohw.go.kr/react/al/sal0301vw.jsp?PAR_MENU_ID=04&MENU_ID=0403&page=1&CONT_SEQ=354112
